# Performance of Olive-Pomace Oils in Discontinuous and Continuous Frying. Comparative Behavior with Sunflower Oils and High-Oleic Sunflower Oils

**DOI:** 10.3390/foods10123081

**Published:** 2021-12-11

**Authors:** Francisca Holgado, María Victoria Ruiz-Méndez, Joaquín Velasco, Gloria Márquez-Ruiz

**Affiliations:** 1 Instituto de Ciencia y Tecnología de los Alimentos y Nutrición, Consejo Superior de Investigaciones Científicas, 28040 Madrid, Spain; f.holgado@ictan.csic.es; 2 Instituto de la Grasa, Consejo Superior de Investigaciones Científicas, 41013 Sevilla, Spain; mvruiz@ig.csic.es (M.V.R.-M.); jvelasco@ig.csic.es (J.V.)

**Keywords:** frying, high-oleic sunflower oil, olive-pomace oil, polar compounds, polymers, sunflower oil

## Abstract

Frying performance of olive-pomace oils (OPOs) as compared to sunflower oils (SOs) and high-oleic sunflower oils (HOSOs) was studied in discontinuous frying (DF) and continuous frying (CF) for the first time. DF is used in household, restaurants and frying outlets, while CF is used in the food industry. Oil alteration during frying was determined by measurements of polar compounds (PC) and polymers. Fried potatoes were analyzed for oil absorption and alteration, color, and evaluated in an acceptability test. Results for DF showed that all SOs reached 25% PC at the 9th frying operation (FO), whereas HOSOs did between the 17–18th FO and variable results were found for OPOs since initial levels of diacylglycerols were different. Rates of formation of PC or polymers were the lowest for OPOs, thus showing the best performance in DF. Specifically for PC, relative rates of formation were 1.00–1.11, 2.46–2.71 and 1.37–1.41 for OPOs, SOs and HOSOs respectively. In CF, OPOs and HOSOs behaved similarly and better than SOs, although none reached 25% PC after 40 FO. The good performance of OPOs can be attributed to the high monounsaturated-to-polyunsaturated ratio, in common with HOSOs, and the additional positive effect of minor compounds, especially β-sitosterol and squalene.

## 1. Introduction

Oils obtained from olives, including virgin olive oils, olive oils and olive-pomace oils, are key components of the Mediterranean diet and stand out for their high content of oleic acid and low amount of polyunsaturated fatty acids thereby presenting great stability and suitability for frying [1]. Olive-pomace oil is obtained from the olive paste generated as a byproduct in the extraction of virgin olive oil. Moisture is eliminated from the wet olive paste and the remaining oil is extracted with *n*-hexane, refined and mixed with virgin olive oil to obtain the commercialized olive-pomace oil. Virgin olive oil contains a pool of minor compounds of strong antioxidant activity, among which phenolic compounds are particularly relevant. However, except for lignans [2], phenolic compounds are drastically removed during the refining process of all crude olive oils and these are practically absent in olive-pomace oil [3]. Once the refined olive-pomace oil is mixed with virgin olive oil to obtain the commercial olive-pomace oil, contribution of phenolic compounds slightly increases. The refining process does not greatly affect the content of other minor components that could improve oil stability such as squalene. In addition, the high content in oleic acid and the presence of minor compounds with beneficial biological activity contribute to the health properties of olive-pomace oils [4]. Specifically, erythrodiol and uvaol have been reported to protect from cardiac hypertrophy [5] and to exert antiatherogenic [6], anti-inflammatory [7], anti-hypertensive [8] and neuroprotective effects [9]. As to aliphatic fatty alcohols, also characteristic of OPOs, studies have shown anti-inflammatory activity [10] and improvement of lipoprotein profile [11]. For all these reasons olive-pomace oil can be an excellent alternative oil for frying. However, the frying performance of olive-pomace oils in comparison with other oils has been scarcely studied so far. To our knowledge, only the following few studies have been reported.

Tekin and coworkers [12] compared hazelnut, olive-pomace, grapeseed and sunflower oils and found satisfactory thermal performance of hazelnut and olive-pomace oil. As to minor components, even when tocopherols were at trace amounts in the olive-pomace oil, the presence of phenolic compounds and other compounds not described in the compositional data could have explained its good performance. Similarly, Bulut and Yilmaz [13] showed that, in general, accumulation of polar compounds in sunflower oil samples was higher at frying conditions than in refined olive-pomace oil samples. Certainly, the fatty acid composition of sunflower oils is much more susceptible to degradation than that of olive-pomace oils [1,14,15]. The high level of tocopherols in the sunflower oil used in the study of Bulut and Yilmaz did not seem to exert sufficient protection to compensate the effect of the high degree of unsaturation [13]. Unfortunately, precise information on other minor compounds present in the oils was not provided. Giuffrè and coworkers [16] evaluated variations in chemical parameters of vegetable oils during heating at 180 and 220 °C. They reported data that suggested better performance of extra virgin olive oil and palm oil than olive-pomace oil, but neither polar compounds nor polymers, the most recommended measurements of frying oil alteration, adopted in countries where the level of alteration is regulated and limited for human consumption, were determined [17]. Giuffrè and coworkers have also reported recently that the major volatile compound detected in fresh olive-pomace oil was decanal, followed by (*E*)-2-hexenal, (*E*)-2-undecenal and nonanal [18]. In the same work, they studied changes of volatile profiles of olive-pomace oil at 180 and 220 °C for the first time, as well as in extra virgin olive oil and palm oil. Other publications on the use of olive-pomace oils in frying have focused on improving their stability by adding coconut oil [19,20], squalene [21] or olive leaf extracts [22].

Among the main factors influencing oil degradation during frying are intrinsic factors, namely, the oil composition, which includes the degree of unsaturation, content of free fatty acids and antioxidants; and external factors such as the length of heating, temperature and surface-to-oil volume ratio, these latter clearly depending on the type of frying, i.e., discontinuous or continuous. In discontinuous frying (domestic frying, frying in restaurants and frying outlets), temperature, length of heating and the periods that the oil remains at high temperature in the absence of food greatly contribute to enhancing alteration. However, in continuous frying (industrial frying), the food is always present in the fryer protecting the oil from the air and the surface-to-oil volume ratio is kept constant by continuous addition of fresh oil [14].

In this study, olive-pomace oils were compared to sunflower oils, including for the first time high-oleic sunflower oils. Sunflower oils are widely used in the food industry and high-oleic seed variants were developed to improve the fatty acid composition in nutritional terms and increase stability for frying [15]. Oils performance was tested under both discontinuous and continuous frying conditions, the latter not used so far in studies on olive-pomace oils. Oils were thoroughly characterized including analyses of minor compounds, and follow-up of oil alteration during frying was carried out by measurements of polar compounds and polymers in both the oils and the fried potatoes. Additionally, the fried potatoes were analyzed for oil absorption, color and evaluated in a preliminary acceptability test.

## 2. Materials and Methods

### 2.1. Materials and Samples

Reagents and standards were purchased from Sigma-Aldrich (St. Louis, MO, USA). Solvents were purchased from Panreac SA (Barcelona, Spain). All reagents and solvents used were of analytical quality. Olive-pomace oils (OPOs), sunflower oils (SOs) and high-oleic sunflower oils (HOSOs) from three different batches each and without tocopherols nor dimethylpolysiloxane added were supplied by ACESUR (Acesur, SA, Sevilla, Spain). OPOs were produced in Spain and all oils were refined by ACESUR and used in this study right after receipt. Fresh in-season potatoes (Agria variety, origin: Spain) were purchased in a local supermarket. Moisture content (% on potato weight) was 78.9 ± 0.7.

### 2.2. Frying Experiments

OPOs, SOs and HOSOs were used for frying operations under discontinuous and continuous conditions, using one-liter fryers (Moulinex AF2200, GroupSEB, Ecully, France). Potatoes were peeled, cut into homogeneous sticks (1 × 1 × 6 cm), washed with water and wrapped in absorbent kitchen paper before frying. Discontinuous and continuous frying experiments were carried out following previous procedures with slight modifications [23]. Temperature was set at 175 °C in both cases and an initial heating period of 20 min was considered. The temperature was controlled by a K-type thermocouple coupled to a recorder, so that each frying operation started at 175 ± 3 °C. Batches of 100 g potatoes were used in each frying operation.

In discontinuous frying, potatoes were fried during 10 min and intervals of 20 min were established between frying operations. After frying, the basket was shaken and held drained for 1 min to remove excess oil. The endpoint of frying operations was established when the total content of polar compounds reached 25%. The experiments were carried out in three consecutive days (7 frying operations each day).

In continuous frying, two baskets were used to maintain the presence of food in the fryer during the whole heating period. Potatoes were fried during 10 min and 60 mL of oil were added between every five frying operations to maintain the same amount of oil in the fryer. The experiments (20 frying operations) were carried out continuously the same day.

Samples of frying oils and fried potatoes were taken after each frying operation and stored at −30 °C until further analyses. Samples of fried potatoes taken for color measurement and sensory evaluation were analyzed right after frying.

### 2.3. Quality and Characterization Parameters of Fresh Oils

The quality and characterization parameters were evaluated according to standard methods.

Free fatty acidity was determined according to method COI/T.20/Doc. 34/Rev. 1-2017 [24].

Peroxide value was determined according to method COI/T.20/Doc. 35/Rev. 1-2017 [24].

Oil Stability Index (OSI) was determined using a Rancimat apparatus at 100 °C following AOCS Official Method Cd-12b-92 [25].

Smoke point was determined following AOCS Official method Cc 9a-48 [25].

Fatty acid composition was determined by gas-liquid chromatography after oil derivatization into fatty acid methyl esters following IUPAC methods 2301 and 2302 [26].

Tocopherols were determined by high-performance liquid chromatography with fluorescence detector following ISO method 9936:2016 [27].

Unsaponifiable matter was determined with diethyl ether following ISO method 3596:2000 [27].

Composition and content of sterols and triterpene dialcohols were determined by gas-liquid chromatography according to method COI/T.20/Doc. no. 30/Rev. 1-2017 [24].

Aliphatic alcohols were determined by gas-liquid chromatography according to the European Union Regulation [28].

Biophenols were determined by HPLC according to method COI/T.20/Doc. nº 29/rev. 1. 2017 [24]. Original chromatograms recorded at 280and 335 nm are shown in Supplementary Materials (Figures S1 and S2) to illustrate analyses of the olive-pomace oil with the lowest content of phenolic compounds (OPO2).

Squalene was determined by gas-liquid chromatography following AOCS Official Method Ch 8–02 [25].

### 2.4. Total Content and Distribution of Polar Compounds

The amount of polar compounds was determined by silica column chromatography following IUPAC method 2507 [26]. The polar fractions were further analyzed by high-performance size-exclusion chromatography, as previously described [29], in order to quantitate oxidized triacylglycerol monomers, triacylglycerol dimers, triacylglycerol polymers, diacylglycerols and free fatty acids.

### 2.5. Color of Oils and Fried Potatoes

Color measurements were made at room temperature using a HunterLab Spectrophotometer 150 CM-3500D (Hunter Associates laboratory, Stamford, CT, USA) with illuminant D65. The color space system used was CIE-L*a*b*. L* value represents lightness-darkness dimension (0–100), a* value represents red-green dimension (−120 to 120), and b* value represents yellow-blue dimension (−120 to 120) [30]. Three samples of each oil were evaluated and measurements in fried potatoes were performed in three sticks from the same frying batch, after the 4 discontinuous frying operation, presenting the lightest, intermediate and darkest color.

### 2.6. Acceptability Test of Fried Potatoes

Sensory analyses were performed by 16 regular consumers of fried potatoes. (9 females and 7 males with age range of 30–55 years old). The attributes evaluated were texture (crispiness), oiliness, taste, color and global appreciation. A 9-point hedonic scale was used for taste and global appreciation where 0 indicated “dislike extremely” and 9 indicated “like extremely” [31]. Texture, oiliness and color were evaluated in a 9-point scale where 0 indicated “not crispy”, “not oily” and “very light”, respectively, and 9 indicated “very crispy”, “very oily” and “very dark”, respectively. The sensory evaluation sheet also included the definition of each attribute, as shown in Supplementary Materials (Figure S3). Before starting the sensory analysis, the panellists were familiarized with the scoring method and attributes to be evaluated. The sensory analyses were performed 3–5 min after removing samples from the fryers, while still hot. Sensory analyses were performed with fried potatoes obtained from the 3rd–6th discontinuous frying operations. Three fried potato sticks for each type of frying oil were presented in a large white plate, and the sensory evaluation was carried out in individual booths.

### 2.7. Lipid Extraction of Fried Potatoes

Fried potatoes were frozen, freeze-dried and ground. Their lipids were obtained by Söxhlet extraction with hexane for 6 h according to method UNE 55-062-80 [32].

### 2.8. Analysis of Oils and Oils Extracted from Fried Potatoes during Frying

Total polar compounds were measured during frying with the Testo-270 oil tester (Testo AG, Lenzkirch, Germany). The probe was immersed into the hot oil and data were collected after 2–3 s while gently stirring the oil for uniform measurement. Polymers were determined directly by high-performance size-exclusion chromatography following IUPAC method 2508 [26].

### 2.9. Statistical Analysis

Characterization and quality analyses of fresh oils were performed in triplicate and data were expressed as means ± standard deviations. The analyses of the frying experiments were also performed in triplicate and data were expressed as means ± standard deviations. One-factor ANOVA was applied using 24.0 SPSS Statistics program (SPSS Inc., Chicago, IL, USA). Tukey’s test was used for comparisons between means and significance was defined at *p* < 0.05.

## 3. Results and Discussion

### 3.1. Characterization and Quality Parameters of Fresh Oils

Table 1 and Table 2 show quality and characterization parameters of the fresh oils.

Free acidity and peroxide value were within the range normally found for refined oils [28,33]. In OPOs, values for free acidity were 0.12–0.21% oleic acid and peroxide values were 2.4–3.0 meq O_2_/kg oil, below the limits established for OPOs, i.e., ≤1% and ≤15 meq O_2_/kg oil, respectively [28]. In all sunflower oils, values for free acidity were 0.05–0.06% oleic acid and peroxide values were 3.2–6.7 meq O_2_/kg oil, likewise below the limits established, i.e., ≤0.2% and ≤10 meq O_2_/kg oil, respectively [33].

Oil Stability Index was over three-fold higher in HOSOs and OPOs than in SOs. Such an index does not provide information on the oil frying performance but gives useful comparative data to rank oils according to their oxidative behavior at low or moderate temperatures. Smoke points were similar in SOs and HOSOs and lower in OPOs. This is consistent with the values reported for sunflower oils and refined olive-pomace oils, 233 and 185 °C, respectively [13].

As expected, the fatty acid compositions reflected the high proportion of oleic acid in OPOs and HOSOs in contrast with that in SOs. Data for SOs and HOSOs were within the range established for *Codex Standard for Named Vegetable Oils* corresponding to sunflowerseed oils and sunflowerseed oils—high oleic acid, respectively [33], and similar to the values normally found in the literature [15,34,35]. As to OPOs, the fatty acid composition of the oils tested was within the limits established, with oleic acid as the major fatty acid (72.02–73.80%), followed by palmitic acid (11.03–11.45%) and linoleic acid (9.54–11.12%) [28]. *Trans* fatty acids were below 0.32%, much lower than contents reported for other OPOs [19].

Table 2 lists composition of minor components of the unsaponifiable fraction. The total content of minor components was higher for OPOs, and their composition was different compared to SOs and HOSOs, especially due to the occurrence of squalene, triterpenic and aliphatic alcohols, and residual amounts of phenolic compounds and triterpenic acids. However, all oils presented similar contents of total sterols and tocopherols.

Total sterols levels were relatively high in all oils. As expected, the sterol composition of OPOs differed from those of the sunflower oils, showing an elevated proportion of β-sitosterol (85.8–88.6%). Other compounds, such as triterpenic dialcohols, aliphatic alcohols and squalene, were only present, and in considerable amounts, in OPOs. However, the content of triterpenic acids, otherwise relevant in virgin olive oils, was just residual in OPOs because these are practically lost during refining of crude olive-pomace oils [36]. Among the OPOs, OPO1 was particularly rich in sterols and aliphatic alcohols, whereas squalene was especially abundant in OPO2. It is well known that contents and differences in composition of minor compounds in OPOs greatly depend on the storage time of the wet olive paste, generally the longer the higher [3,37,38]. In addition to the variability in crude oils, differences in refining conditions may also contribute to the differences found between OPOs [3].

Tocopherol levels were approximately in the range 300–500 mg/kg in all oils and α-tocopherol was by far the most abundant tocopherol. Other phenolic compounds were only present in OPOs, although in very low amounts, ranging from 16–33 mg/kg, because these are practically lost during refining [3]. Among the OPOs, OPO1 presented higher contents in tocopherols and total phenolic compounds.

### 3.2. Total Content and Distribution of Polar Compounds in the Fresh Oils

Table 3 shows the total content and composition of polar compounds in the fresh oils. OPOs and HOSOs showed the highest and lowest levels of total polar compounds, respectively. However, the polar fraction in OPOs did not comprise substantial levels of oxidation compounds, but diacylglycerols, which remain after refining as indicators of hydrolytic alteration of the crude oils [39]. Such hydrolytic reactions are attributed in part to the enzymatic action occurring during long storage of wet olive paste in ponds [40].

Regarding oxidation compounds, levels were similarly low for all oils, although oxidized triacylglycerol monomers and dimers were significantly higher in SOs and OPOs, respectively, than in HOSOs.

### 3.3. Discontinuous Frying Experiments

Follow-up of oil alteration during frying was carried out by two measurements, polar compounds and polymers. Both are widely accepted and adopted in a number of countries to establish limits for human consumption, specifically, 24–27% polar compounds and 10–16% polymers [17].

**Figure 1:**
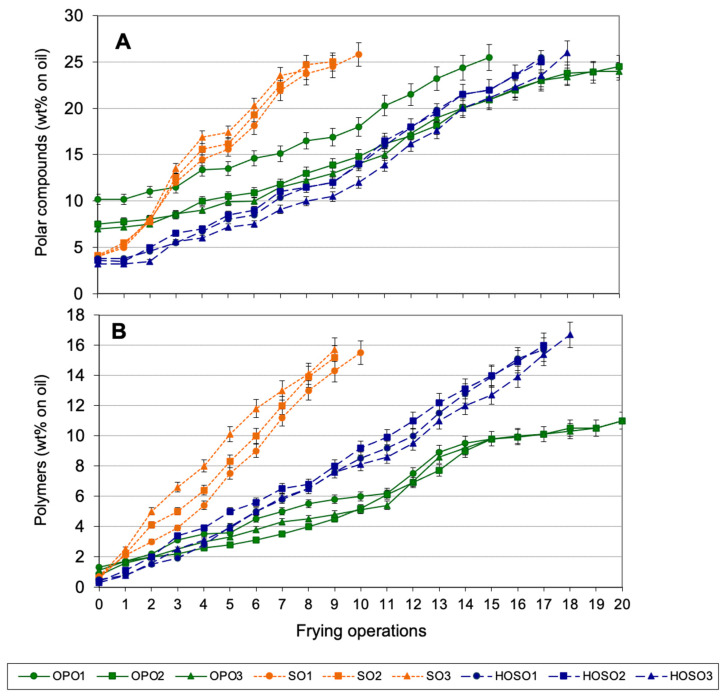
Polar compounds (**A**) and polymers (**B**) in used frying oils during discontinuous frying. Abbreviations: OPO, olive-pomace oil; SO, sunflower oil; HOSO, high-oleic sunflower oil. Each value is the mean ± SD of three determinations. Samples were analyzed in all frying operations until oils reached 25% polar compounds.

Figure 1 shows the levels of total polar compounds (A) and polymers (B) in oils during discontinuous frying experiments. All SOs reached 25% polar compounds at the 9th frying operation and all HOSOs did between the 17–18th frying. The endpoint for OPO2 and OPO3 was the longest, at the 21st frying operation, in contrast with OPO1, at the 15th frying, since its starting level of polar compounds was the highest. 

Given that all fresh OPOs contained the highest levels of polar compounds due to the diacylglycerol contribution, polymer analysis offered a more objective tool to compare oils degradation during frying. Polymers include those compounds predominantly formed during frying, i.e., triacylglycerol dimers, trimers and higher oligomers. As shown in Figure 1B, all oils started from similarly low polymer levels and SOs, followed by HOSOs, accumulated higher amounts during frying as compared to OPOs.

In most countries where alteration of frying fats and oils is regulated for human consumption the level of 24–27% polar compounds (on total oil weight) is the limit established. Some countries consider the level of polymers instead or together with the level of polar compounds. For example, used frying oils cannot surpass 25% polar compounds (on total oil weight) or 10% polymers (on total oil weight) in Belgium, while regulation in Netherlands only considers polymers as measurement to control alteration and establishes a limit of 16% [17].

Formation of total polar compounds and polymers followed a zero-order kinetic for all the oils under the conditions applied, in agreement with results obtained in previous studies [41]. Table 4 summarizes the main parameters for linear regression.

Linear correlation coefficients were higher than 0.98. The relative rates of formation of polar compounds and polymers have been included, assuming value 1 for the oil presenting the lowest rate. For both measurements, and especially for polymers, the rate of formation was the lowest in OPOs. For example, polymers formed three times slower in OPOs than in SOs and almost twice slower than in HOSOs.

Table 5 includes polar compound distribution in oil samples withdrawn in the last frying operation, when total amounts reached approximately 25%.

As can be observed, hydrolytic compounds remained practically at the same levels as those found in the fresh oils (Table 3) and, among the groups of oxidation compounds, the greatest increments were found for triacylglycerol polymers, which were significantly higher for SOs and HOSOs than for OPOs.

**Figure 2:**
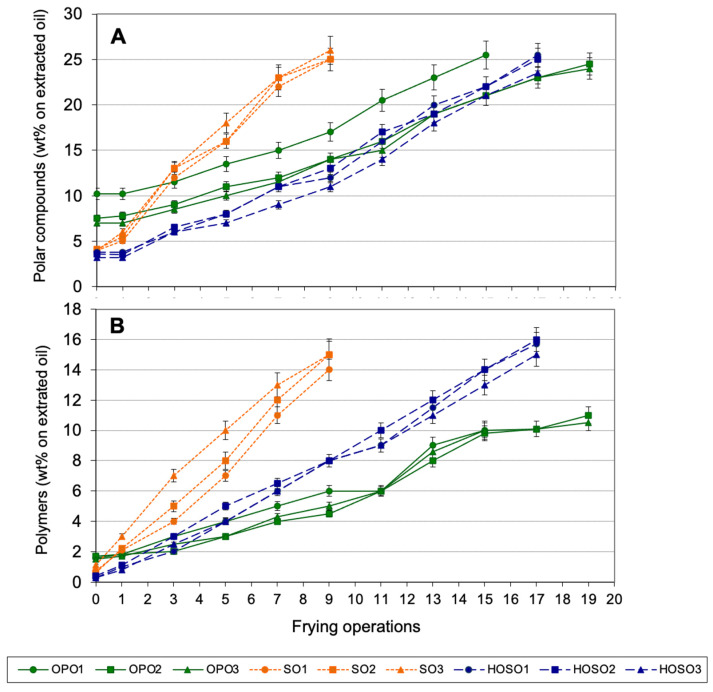
Polar compounds (**A**) and polymers (**B**) in oils extracted from fried potatoes during discontinuous frying. Abbreviations: OPO, olive-pomace oil; SO, sunflower oil; HOSO, high-oleic sunflower oil. Each value is the mean ± SD of three determinations. Samples were analyzed every other frying operation starting in the first one and until the oils extracted from fried potatoes reached 25% polar compounds.

Figure 2 shows the levels of total polar compounds (A) and polymers (B) in oils extracted from fried potatoes during discontinuous frying experiments.

Results showed no significant differences between the level of alteration in the used frying oil and that in the oil absorbed by the fried potatoes in the samples analyzed. Therefore, no preferential absorption of polar compounds was observed, as previously reported [23,41,42]. This also means that the degradation of the used frying oil was representative of that in the fried food.

As commented in the Introduction, there is scant information published on the frying performance of olive-pomace oils. In contrast, a plethora of studies have been reported on frying experiments comparing virgin olive oils, olive oils, sunflower oils and other seed oils, as we already discussed in a review [1]. Virgin olive oils are highly resistant to alteration during frying mainly because of their high monounsaturated-to-polyunsaturated fatty acid ratio and the antioxidant activity of phenolic compounds [43]. However, frying behavior of virgin olive oils depends considerably on the olive variety [44] and olive ripening degree [45,46].

Even though phenolic compounds are drastically reduced during refining, refined olive oils have also shown to be less prone to alteration during frying than refined unsaturated seed oils, such as soybean, sunflower and corn oils [47,48]. As to comparisons between olive oils and high-oleic sunflower oils, Dobarganes and coworkers carried out studies in original oils, oils stripped of antioxidants and the latter with added tocopherols, and showed that the better results found for olive oil were essentially attributed to the protective effect of antioxidants other than tocopherols present in olive oils [49,50].

In the present study, the best frying performance found for OPOs as compared to SOs is expected from the differences in the fatty acid composition, as it is well known that oleic acid is more stable than linoleic acid. Compared to HOSOs, the better frying performance of OPOs does not seem to be attributable either to the fatty acid composition, since they showed similar monounsaturated-to-polyunsaturated fatty acid ratio, or to tocopherols, since their concentrations were similar or lower in OPOs, or to phenolic compounds, present in very low amounts in OPOs (Table 2). Instead, it could be related to the potential protective effects of other minor compounds such as squalene and β-sitosterol. It has been reported that sterols, especially β-sitosterol, in higher concentrations in OPOs than in SOs or HOSOs, improve oil stability during frying [51,52]. However, the antioxidant mode of action of phytosterols is not clear. Their conversion into steradienes at frying temperatures, whose conjugated diene system could ultimately reduce polymer formation, has been proposed [52]. With respect to squalene in vegetable oils, it is only found in considerable amounts in olive-extracted oils. It appears that squalene does not exert a protective effect alone, as it was observed in the present study, since levels in OPO2 doubled those in OPO1 and OPO3 but the frying performance was similar for all OPOs. Otherwise, its positive influence on frying seems to be due to its combined action with tocopherols as secondary antioxidants [53]. Thus, it has been suggested that α-tocopherol can be regenerated from the tocopheroxyl radical by squalene [54].

### 3.4. Continuous Frying Experiments

**Figure 3:**
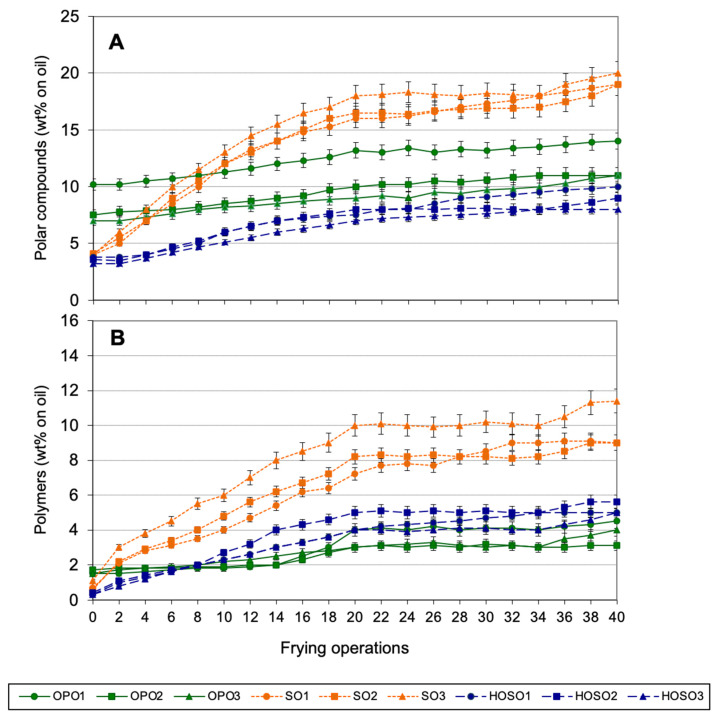
Polar compounds (**A**) and polymers (**B**) in used frying oils during continuous frying. Abbreviations: OPO, olive-pomace oil; SO, sunflower oil; HOSO, high-oleic sunflower oil. Each value is the mean ± SD of three determinations. Samples were analyzed in all frying operations (1–40).

Figure 3 shows the levels of total polar compounds (A) and polymers (B) in oils during continuous frying experiments. None of the oils reached 25% polar compounds after 40 frying operations. As expected, continuous frying led to much lower oil alteration than discontinuous frying, due to addition of fresh oil and the absence of non-heating periods but, most importantly, the protection towards the air entrance conferred by the constant presence of food in the fryer [14]. As to polymers (Figure 3B), there were no relevant differences between HOSOs and OPOs and both types of oil showed the best performance.

The remarkable differences found in oil alteration during frying between discontinuous and continuous processes are consistent with previous results obtained for SO and HOSO in similar frying experiments wherein, after 6-h frying, the amounts of polar compounds in both oils were approximately twice as much in discontinuous as compared to the continuous process [23]. Likewise, Totani and workers compared frying performance of canola oil under discontinuous and continuous conditions and reported values over 10% polar compounds and approximately 6%, respectively, after the same frying time (6 h) [55].

As to comparisons between SO and HOSO, results obtained by Jorge and coworkers showed that, after 32 continuous frying operations, SO and HOSO reached 11.7 and 7.0% polar compounds, respectively, which are values consistent with those obtained in the present study [23].

Even though the frying continuous experiments carried out in the present work just simulated industrial frying, a plateau effect is observed, similarly to that occurring in industrial continuous fryers as a result of the continuous reposition of the oil absorbed by the food with fresh oil [56].

Total polar compounds and polymers were also analyzed in oils extracted from fried potatoes during continuous frying experiments. Results are not shown for the sake of brevity since, similarly to what was found in discontinuous experiments, there were no significant differences between the level of alteration in the used frying oil and that in the oil absorbed by the fried potatoes.

### 3.5. Evaluation of Fried Potatoes from Discontinuous Experiments

Table 6 includes color evaluation in fresh oils and fried potatoes as well as oil contents in fried potatoes. As expected, color parameters L*, a* and b* were significantly different in OPOs, derived from olives, from those in SOs and HOSOs, being OPO1 the darkest. In all OPOs, as compared to SOs and HOSOs, the chromatic component L* (lightness) was lower, a* was higher in a negative direction, denoting greenness, and b* (yellowness value) was substantially higher in a positive direction. However, these differences were not noted in the fried potatoes, which showed similar values regardless of the oil used and were close to the values expected for fried potatoes of the Agria variety [57].

As to the oil absorbed in the fried potatoes, there were no significant differences between the oils used, and the contents found were about 12%. It is well-known that besides temperature and time of frying, the specific characteristics of the potatoes (moisture, surface microstructure, porosity, density, etc.) and the shape, size, surface-to-volume ratio and surface roughness obtained in potato preparation are relevant variables determining the amount of oil absorbed [58,59]. Therefore, variable results in oil contents can be found in fried potato sticks prepared from fresh potatoes, usually ranging from 8–12% [23,30].

In contrast with the results obtained in this work, some studies have shown that the type of oil used may have influence on oil absorption in fried potatoes although this effect is normally associated with significant differences in oil viscosity in that the higher the oil viscosity, the greater the oil accumulation on the surface of the fried food and so the oil penetration during the cooling period [60,61].

Sensory analyses were carried out by 16 regular consumers of fried potatoes to preliminarily evaluate the level of acceptability of fried potatoes using OPOs (Table 7). In agreement with color evaluation (Table 6), sensory analyses did not show differences in color attribute in fried potatoes. Also, the lack of differences noted by panellists in terms of oiliness was consistent with the results found for oil absorption, which did not depend on the oil used (Table 6). Neither of the other attributes tested, i.e., texture and taste, as well as global appreciation, showed significant differences between the potatoes fried with different oils.

## 4. Conclusions

Starting from sunflower, high-oleic sunflower and olive-pomace oils with good quality parameters, this study has allowed comparison of frying performance in both discontinuous and continuous conditions. One important conclusion is that when comparing oils with different starting contents of polar compounds due to the residual presence of diacylglycerols after refining, as it occurred here with OPOs, polymer determination is more appropriate to evaluate the stability at frying conditions. In this study, OPOs and HOSOs showed much better frying performance than SOs. Although OPOs and HOSOs behaved similarly in continuous frying simulating industrial preparation of fried potatoes, better results were obtained with OPOs in discontinuous frying, a process normally used in household, restaurants and frying outlets, and performed under more adverse conditions for oil stability than those used in continuous frying. The good performance of OPOs can be attributed to the high monounsaturated-to-polyunsaturated fatty acid ratio, in common with HOSOs, and the additional positive effect of the pool of minor compounds, especially β-sitosterol and squalene.

## Figures and Tables

**Table 1:** Characterization and quality parameters of fresh oils.

	OPO1	OPO2	OPO3	SO1	SO2	SO3	HOSO1	HOSO2	HOSO3
Acidity (% oleic acid)	0.21 ± 0.03 d	0.12 ± 0.02 c	0.12 ± 0.01 c	0.06 ± 0.01 b	0.05 ± 0.01 ab	0.06 ± 0.01 b	0.05 ± 0.01 ab	0.05 ± 0.01 ab	<0.05 a
Peroxide value (meq O_2_/kg oil)	3.0 ± 0.6 a	2.8 ± 0.5 a	2.4 ± 0.5 a	5.7 ± 1.1 bc	6.6 ± 1.3 c	7.6 ± 1.2 c	3.8 ± 0.8 ab	3.6 ± 0.6 ab	3.2 ± 0.6 a
Oxidative Stability Index (h)	40.7 ± 1.5 cd	44.0 ± 1.9 d	42.8 ± 2.1 d	11.6 ± 1.1 a	10.9 ± 1.0 a	10.9 ± 0.9 a	36.7 ± 1.2 bc	32.9 ± 1.3 b	41.0 ± 1.5 d
Smoke point (°C)	190 ± 2 a	194 ± 2 a	192 ± 3 b	230 ± 1 c	234 ± 2 c	233 ± 2 c	233 ± 3 c	233 ± 2 c	233 ± 2 c
Fatty acid composition (%)									
C16:0	11.03 ± 0.44 c	11.44 ± 0.31 c	11.45 ± 0.22 c	6.45 ± 0.15 b	6.40 ± 0.11 b	6.59 ± 0.23 b	4.51 ± 0.20 a	4.75 ± 0.18 a	4.22 ± 0.21 a
C16:1	0.85 ± 0.04 b	0.91 ± 0.05 b	0.83 ± 0.06 b	0.13 ± 0.04 a	0.10 ± 0.03 a	0.10 ± 0.04 a	0.18 ± 0.03 a	0.14 ± 0.01 a	0.14 ± 0.02 a
C18:0	3.16 ± 0.14 abc	2.92 ± 0.10 ab	2.86 ± 0.12 a	3.40 ± 0.09 cd	3.53 ± 0.15 cd	3.71 ± 0.20 d	3.23 ± 0.14 abc	3.28 ± 0.12 bc	3.27 ± 0.13 bc
C18:1	73.80 ± 0.89 e	72.02 ± 0.65 d	72.06 ± 0.60 d	32.16 ± 0.38 c	30.04 ± 0.29 b	28.38 ± 0.20 a	80.35 ± 0.70 g	78.04 ± 0.67 f	81.21 ± 0.44 g
C18:2	9.54 ± 0.26 a	11.00 ± 0.16 b cd	11.12 ± 0.10 cd	56.56 ± 0.77 e	58.70 ± 0.80 f	60.00 ± 0.98 f	10.15 ± 0.10 abc	12.31 ± 0.12 d	9.56 ± 0.10 ab
C18:3	0.62 ± 0.04 c	0.40 ± 0.03 b	0.45 ± 0.02 b	0.08 ± 0.01 a	0.08 ± 0.01 a	0.09 ± 0.01 a	0.08 ± 0.01 a	0.08 ± 0.01 a	0.09 ± 0.00 a
C20:0	0.46 ± 0.04 d	0.19 ± 0.02 a	0.23 ± 0.02 a	0.31 ± 0.02 b	0.29 ± 0.03 b	0.29 ± 0.04 b	0.35 ± 0.03 c	0.33 ± 0.04 c	0.36 ± 0.02 c
C20:1	0.37 ± 0.04 d	0.16 ± 0.01 a	0.18 ± 0.01 a	0.26 ± 0.02 b	0.24 ± 0.02 b	0.23 ± 0.01 b	0.31 ± 0.02 c	0.30 ± 0.0.03 c	0.31 ± 0.02 c
C22:0	0.17 ± 0.02 b	0.05 ± 0.01 a	0.05 ± 0.00 a	0.65 ± 0.03 c	0.61 ± 0.02 c	0.62 ± 0.04 c	0.84 ± 0.05 d	0.78 ± 0.03 d	0.83 ± 0.05 d
Total *trans* fatty acids	0.21 ± 0.01 b	0.32 ± 0.01 c	0.29 ± 0.03 bc	0.10 ± 0.01 a	0.12 ± 0.01 a	0.08 ± 0.01 a	0.20 ± 0.01 b	0.19 ± 0.02 b	0.16 ± 0.02 b

Abbreviations: OPO, olive-pomace oil; SO, sunflower oil; HOSO, high-oleic sunflower oil. Means ± SD (*n* = 3). Different letters in the same row indicate significant differences according to Tukey’s test at *p <* 0.05.

**Table 2:** Characterization of minor compounds in fresh oils.

	OPO1	OPO2	OPO3	SO1	SO2	SO3	HOSO1	HOSO2	HOSO3
Unsaponifiable matter (wt.% on oil)	1.28 ± 0.15 cd	1.32 ± 0.10 cd	1.49 ± 0.08 d	1.08 ± 0.13 bc	0.85 ± 0.08 ab	0.83 ± 0.07 ab	0.77 ± 0.06 a	0.78 ± 0.05 a	0.95 ± 0.10 ab
Sterols (wt.% on total)									
Cholesterol	0.1	0.1	0.1	0.1	0.1	0.1	0.1	0.1	0.1
Brassicasterol	<0.1	<0.1	<0.1	0.0	<0.1	<0.1	<0.1	<0.1	<0.1
Campesterol	2.9	3.0	3.1	9.5	8.9	8.9	9.2	9.2	9.9
Stigmasterol	0.8	1.0	1.0	7.5	7.7	7.7	7.8	7.7	8.6
β-Sitosterol	88.6	87.5	85.8	54.6	55.8	55.5	53.3	53.0	55.2
∆7-Stigmastenol	0.4	0.5	0.4	14.7	14.3	14.5	15.6	15.3	13.6
24-Methylen cholesterol	0.2	0.1	0.1	0.1	0.2	0.1	0.1	0.1	0.2
Campestanol	0.1	0.1	0.1	0.1	0.1	0.1	0.2	0.1	0.1
∆7-Campesterol	0.0	0.0	0.0	2.7	2.5	2.5	3.2	3.2	2.8
∆5,23-Stigmastadienol	0.2	0.2	0.1	0.0	<0.1	<0.1	<0.1	<0.1	<0.1
Clerosterol	1.2	1.5	2.1	0.9	0.7	0.7	1.2	1.1	1.0
Sitostanol	1.4	1.7	1.9	0.5	0.5	0.4	0.4	0.5	0.4
∆5-Avenasterol	1.2	2.0	2.0	2.8	2.9	2.9	2.4	2.6	2.5
∆5,24-Stigmastadienol	1.6	1.7	2.4	1.4	1.1	1.3	1.5	1.6	1.1
∆7-Avenasterol	1.4	0.6	0.9	5.1	5.3	5.4	5.1	5.4	4.5
Total (mg/kg oil)	3348 ± 35 f	2756 ± 24 b	2373 ± 15 a	3328 ± 30 f	3152 ± 26 e	3136 ± 36 de	2982 ± 18 c	3066 ± 32 d	3162 ± 35 e
Triterpenic alcohols (Erythrodiol + Uvaol) (mg/kg oil)	579 ± 18 a	647 ± 30 b	648 ± 27 b						
Aliphatic alcohols (C22 + C24 + C26 + C28) (mg/kg oil)	2269 ± 71 b	1677 ± 58 a	1749 ± 39 a						
Squalene (mg/kg oil)	742 ± 26 a	1538 ± 38 b	816 ± 30 a						
Triterpenic acids (Oleanoic acid + Maslinic acid) (mg/kg)	102 ± 12 a	126 ± 18 a	123 ± 15 a						
Tocopherols (mg/kg oil)									
α-Tocopherol	415	350	272	473	493	482	424	413	431
β-Tocopherol	8	11	13	28	28	26	26	25	27
γ -Tocopherol	23	17	16	20	15	11	15	15	16
δ -Tocopherol	<2	<2	<2	<2	<2	<2	<2	<2	<2
Total	446 ± 24 c	378 ± 18 b	301 ± 10 a	521 ± 28 de	536 ± 32 e	519 ± 13 de	465 ± 21 cd	453 ± 19 c	474 ± 18 cd
Phenols (mg/kg oil)									
Hydroxytyrosol	1	<1	<1						
Tyrosol	1	1	<1						
Vanillic acid	<1	<1	<1						
Vanillin	<1	<1	<1						
*p*-coumaric acid	<1	<1	<1						
Hydroxytyrosol acetate	1	<1	<1						
Dialdehydic form of decarboxymethyl oleuropein aglycone	1	2	3						
Tyrosol acetate	<1	<1	<1						
Dialdehydic form of decarboxymethyl ligstroside aglycone	2	1	1						
Pinoresinol	2	2	2						
Cinnamic acid	<1	<1	<1						
1-Acetoxypinoresinol	1	1	1						
Oleuropein aglycone	6	1	1						
Ligstroside aglycone	1	1	2						
Ferulic acid	<1	<1	<1						
Luteolin	1	<1	<1						
Apigenin	<1	<1	<1						
Total polyphenols	15 ± 1 b	8 ± 1 a	10 ± 1 a						
Total orthodiphenols	9 ± 1 b	3 ± 0 a	4 ± 1 a						
Total secoiridoids	9 ± 1 b	5 ± 1 a	7 ± 1 ab						

Abbreviations: OPO, olive-pomace oil; SO, sunflower oil; HOSO, high-oleic sunflower oil. Means ± SD (*n* = 3). Different letters in the same row indicate significant differences according to Tukey´s test at *p <* 0.05.

**Table 3:** Total content and distribution of polar compounds in fresh oils.

	OPO1	OPO2	OPO3	SO1	SO2	SO3	HOSO1	HOSO2	HOSO3
Total polar compounds (% on oil)	10.3 ± 0.1 a	7.5 ± 0.1 b	7.0 ± 0.1 b	4.0 ± 0.1 c	4.1 ± 0.1 c	4.0 ± 0.1 c	3.7 ± 0.1 c	3.6 ± 0.1 c	3.2 ± 0.1 d
									
Oxidized triacylglycerol monomers	1.1 ± 0.1 a	1.2 ± 0.1 a	1.2 ± 0.1a	1.9 ± 0.2 b	2.2 ± 0.1 b	2.1 ± 0.1 b	1.6 ± 0.1 a	1.3 ± 0.1 a	1.4 ± 0.1 a
Triacylglycerol dimers	1.3 ± 0.1 a	0.8 ± 0.1 a	1.0 ± 0.1 a	0.6 ± 0.1 b	0.6 ± 0.1 b	0.6 ± 0.1 b	0.7 ± 0.1 b	0.3 ± 0.1 b	0.3 ± 0.0 b
Diacylglycerols	7.2 ± 0.2 a	5.0 ± 0.1 b	4.4 ± 0.1 b	1.1 ± 0.1 c	1.0 ± 0.1 c	0.9 ± 0.1 c	1.3 ± 0.1 c	1.2 ± 0.1 c	1.1 ± 0.1 c
Monoacylglycerols	0.3 ± 0.0 a	0.1 ± 0.0 b	0.1 ± 0.0 b	nd	nd	nd	nd	nd	nd
Free fatty acids *	0.2 ± 0.1 a	0.1 ± 0.0 a	0.1 ± 0.0 a	0.1 ± 0.1 a	0.1 ± 0.0 a	0.1 ± 0.0 a	0.1 ± 0.0 a	0.1 ± 0.0 a	<0.1

Abbreviations: OPO, olive-pomace oil; SO, sunflower oil; HOSO, high-oleic sunflower oil. Means ± SD (*n* = 3). Different letters in the same row indicate significant differences according to Tukey´s test at *p <* 0.05. * Includes polar unsaponifiable fraction. nd: not detected.

**Table 4:** Kinetic data for the formation of polar compounds and polymers in oils during discontinuous frying.

Oil	*k* PC (%/h)	*r*	Relative Rate	*k* Pol (%/h)	*r*	Relative Rate
OPO1	2.110 ± 0.10	0.986	1.11	1.089 ± 0.04	0.985	1.04
OPO2	1.903 ± 0.08	0.994	1.00	1.074 ± 0.04	0.983	1.03
OPO3	1.961 ± 0.07	0.990	1.03	1.043 ± 0.04	0.983	1.00
SO1	4.687 ± 0.07	0.991	2.46	3.109 ± 0.05	0.995	2.98
SO2	5.055 ± 0.05	0.991	2.66	3.269 ± 0.06	0.998	3.13
SO3	5.158 ± 0.09	0.984	2.71	3.264 ± 0.07	0.997	3.13
HOSO1	2.691 ± 0.05	0.991	1.41	1.874 ± 0.03	0.996	1.80
HOSO2	2.633 ± 0.04	0.993	1.38	1.821 ± 0.02	0.998	1.75
HOSO3	2.602 ± 0.04	0.987	1.37	1.772 ± 0.03	0.995	1.70

Abbreviations: *k*, rate constant; *r*, linear correlation coefficient; PC, polar compounds; Pol, Polymers; OPO, olive-pomace oil; SO, sunflower oil; HOSO, high-oleic sunflower oil. *k* ± SD (*n* = 20 for OPO, *n* = 10 for SO and *n* = 18 for HOSO).

**Table 5:** Total contents and distribution of polar compounds in used frying oils at the last frying operation (with approximately 25% polar compounds).

	OPO1	OPO2	OPO3	SO1	SO2	SO3	HOSO1	HOSO2	HOSO3
Total polar compounds (wt.% on oil)	25.5 ± 0.1 b	26.0 ± 0.2 b	25.7 ± 0.1 b	25.8 ± 0.2 b	25.0 ± 0.1 a	25.7 ± 0.1 b	25.5 ± 0.2 ab	25.0 ± 0.2 a	26.0 ± 0.2 b
Oxidized triacylglycerol monomers	8.1 ± 0.1 a	9.2 ± 0.2 b	8.9 ± 0.2 b	9.0 ± 0.1 b	8.8 ± 0.2 b	9.0 ± 0.1 b	8.6 ± 0.1 b	7.7 ± 0.2 a	8.2 ± 0.1 a
Triacylglycerol polymers *	9.8 ± 0.2 a	11.3 ± 0.1 b	11.2 ± 0.1 b	15.5 ± 0.1 c	15.2 ± 0.1 c	15.7 ± 0.2 d	15.7 ± 0.3 d	16.0 ± 0.2 d	16.7 ± 0.1 d
Diacylglycerols	7.1 ± 0.2 a	5.3 ± 0.3 b	5.2 ± 0.3 b	1.1 ± 0.2 c	0.9 ± 0.1 c	0.9 ± 0.3 c	1.2 ± 0.1 c	1.2 ± 0.3 c	1.1 ± 0.1 c
Monoacylglycerols	0.2 ± 0.0 a	0.2 ± 0.1 a	0.2 ± 0.1 a	nd	nd	nd	nd	nd	nd
Free fatty acids ^§^	0.3 ± 0.1 a	0.2 ± 0.1 a	0.2 ± 0.0 a	0.2 ± 0.1 a	0.2 ± 0.1 a	0.2 ± 0.1 a	0.2 ± 0.1 a	0.2 ± 0.1 a	0.2 ± 0.1 a

Abbreviations: OPO, olive-pomace oil; SO, sunflower oil; HOSO, high-oleic sunflower oil. Means ± SD (*n* = 3). Different letters in the same row indicate significant differences according to Tukey´s test at *p <* 0.05. * Sum of triacylglycerol dimers and higher oligomers. ^§^ Includes polar unsaponifiable fraction. nd: not detected.

**Table 6:** Color parameters of fresh oils and fried potatoes.

	Oils			Fried Potatoes			
	L*	a*	b*	L*	a*	b*	Oil Content (%)
OPO1	92.50 ± 1.90 a	−7.90 ± 0.50 a	31.93 ± 3.10 a	67.63 ± 1.49 a	3.52 ± 0.88 a	31.56 ± 1.95 a	12.0 ± 0.3 a
OPO2	94.38 ± 1.70 b	−6.96 ± 0.51 b	37.50 ± 1.21 b	64.09 ± 2.37 a	4.32 ± 1.31 a	32.18 ± 1.55 a	11.9 ± 0.2 a
OPO3	95.32 ± 1.55 b	−5.37 ± 0.70 b	36.97 ± 1.18 b	65.82 ± 2.38 a	3.61 ± 1.02 a	32.26 ± 1.70 a	12.1 ± 0.6 a
SO1	98.93 ± 3.10 c	−2.11 ± 0.30 c	7.71 ± 1.00 c	66.72 ± 3.01 a	3.64 ± 1.00 a	32.44 ± 1.92 a	12.3 ± 0.5 a
SO2	99.08 ± 2.94 c	−1.72 ± 0.42 c	6.70 ± 1.00 c	65.23 ± 0.28 a	3.18 ± 0.67 a	32.67 ± 1.97 a	12.1 ± 0.3 a
SO3	99.11 ± 2.78 c	−1.74 ± 0.31 c	6.39 ± 1.00 c	65.08 ± 2.09 a	4.33 ± 0.52 a	31.81 ± 2.08 a	11.9 ± 0.7 a
HOSO1	98.91 ± 3.10 c	−1.95 ± 0.50 c	7.31 ± 1.30 c	66.30 ± 1.52 a	2.98 ± 0.47 a	33.84 ± 1.59 a	12.3 ± 0.7 a
HOSO2	98.64 ± 3.40 c	−2.19 ± 0.41 c	8.20 ± 1.10 c	66.50 ± 0.80 a	2.99 ± 0.90 a	34.86 ± 1.85 a	12.1 ± 0.8 a
HOSO3	98.32 ± 2.90 c	−2.30 ± 0.34 c	8.21 ± 0.90 c	66.56 ± 3.75 a	4.44 ± 0.92 a	33.67 ± 2.14 a	11.9 ± 0.3 a

Abbreviations: OPO, olive-pomace oil; SO, sunflower oil; HOSO, high-oleic sunflower oil. Means ± SD (*n* = 3). Different letters in the same column indicate significant differences according to Tukey´s test at *p <* 0.05.

**Table 7:** Sensory properties of fried potatoes.

	Sensory Attribute				
	Texture	Oiliness	Taste	Color	Global Appreciation
OPO1	4.0 ± 1.3 a	4.7 ± 2.3 a	4.7 ± 1.2 a	4.3 ± 0.6 a	5.2 ± 1.2 a
OPO2	4.0 ± 1.9 a	3.9 ± 1.3 a	5.6 ± 1.5 a	4.5 ± 0.9 a	5.4 ± 1.3 a
OPO3	4.4 ± 1.8 a	4.3 ± 1.8 a	5.4 ± 1.5 a	4.5 ± 0.5 a	5.3 ± 1.5 a
SO1	4.3 ± 1.9 a	4.3 ± 1.9 a	5.1 ± 1.5 a	4.6 ± 1.0 a	5.0 ± 1.2 a
SO2	3.8 ± 1.7 a	4.5 ± 1.6 a	4.7 ± 1.4 a	4.3 ± 1.0 a	4.8 ± 1.2 a
SO3	5.1 ± 2.3 a	5.0 ± 1.3 a	5.8 ± 1.1 a	4.4 ± 2.0 a	5.3 ± 1.1 a
HOSO1	3.5 ± 2.1 a	3.8 ± 1.8 a	5.2 ± 1.0 a	3.7 ± 1.2 a	4.9 ± 0.9 a
HOSO2	4.9 ± 2.0 a	4.8 ± 1.7 a	5.4 ± 1.2 a	4.7 ± 1.3 a	5.7 ± 1.3 a
HOSO3	4.7 ± 1.1 a	5.2 ± 1.6 a	5.2 ± 1.8 a	4.5 ± 2.1 a	5.3 ± 1.1 a

Abbreviations: OPO, olive-pomace oil; SO, sunflower oil; HOSO, high-oleic sunflower oil. Means ± SD (*n* = 16). Different letters in the same column indicate significant differences according to Tukey´s test at *p <* 0.05.

## Data Availability

Not applicable.

## Supplementary Materials

The following are available online at https://www.mdpi.com/article/10.3390/foods10123081/s1, Figure S1: Analysis of phenolic compounds (280 nm) in olive-pomace oil (OPO2), Figure S2: Analysis of phenolic compounds (335 nm) in olive-pomace oil (OPO2), Figure S3: Acceptability test evaluation sheet.
