# Supplementary material for: Performance of Olive-Pomace Oils in Discontinuous and Continuous Frying. Comparative Behavior with Sunflower Oils and High-Oleic Sunflower Oils
Source: Foods. 2021 Dec 11;10(12):3081. doi: 10.3390/foods10123081 (PMC8701874; doi:10.3390/foods10123081)
Supplement: Supplementary file 1 [file foods-10-03081-s001.zip › foods-1481554-Supplementary Material/Supplementary Figure S1. OPO2 Phenolic compounds 280 nm.pdf]

Figure S1. Analysis of phenolic compounds (280 nm) in olive-pomace oil (OPO2).

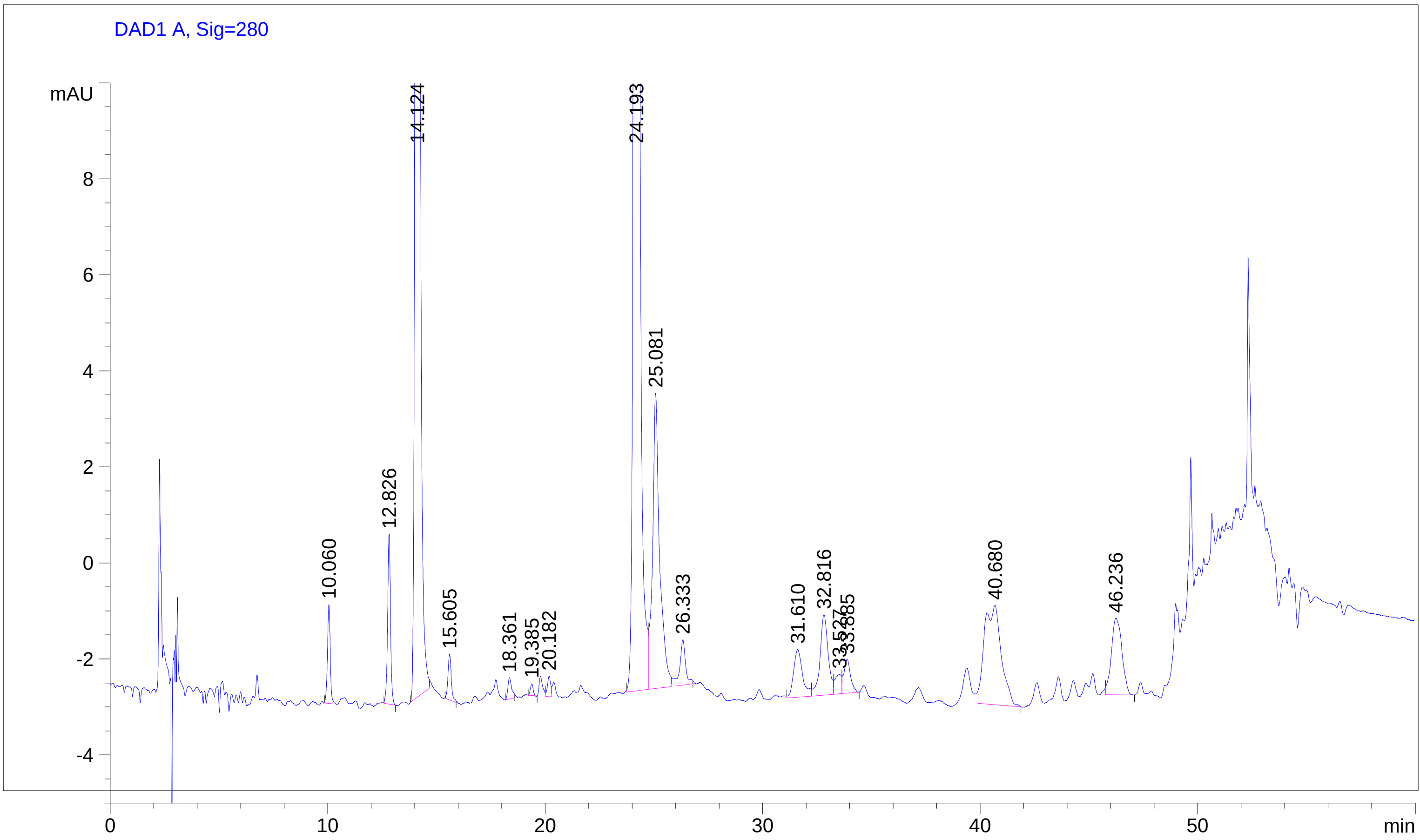

==

Area Percent Report

==

Sorted By : Signal
Multiplier : 1.0000
Dilution : 1.0000
Use Multiplier & Dilution Factor with ISTDs

Signal 1: DAD1 A, Sig=280,16 Ref=550,80, TT

Peak #	RetTime [min]	Type	Width [min]	Area [mAU*s]	Height [mAU]	Area %
1	10.060	BB	0.1351	18.26189	2.06635	0.7200
2	12.826	BP	0.1372	32.59294	3.54811	1.2850
3	14.124	PB	0.1422	1141.78223	120.92918	45.0166
4	15.605	BB	0.1593	10.04263	9.50241e-1	0.3959
5	18.361	PB	0.1719	5.34544	4.45836e-1	0.2108
6	19.385	PP	0.1314	2.18873	2.46866e-1	0.0863
7	20.182	BV	0.1537	4.42900	4.24647e-1	0.1746
8	24.193	BV	0.2099	902.31293	65.03934	35.5752
9	25.081	VB	0.3200	141.40019	6.15636	5.5749
10	26.333	BB	0.2563	16.39130	9.45447e-1	0.6463
11	31.610	BV	0.4263	28.47864	9.99734e-1	1.1228
12	32.816	VV	0.3872	43.84283	1.67594	1.7286
13	33.527	VV	0.2571	8.31255	4.09098e-1	0.3277
14	33.885	VP	0.3201	15.22804	7.10366e-1	0.6004
15	40.680	BP	0.7010	111.68678	2.07165	4.4034
16	46.236	BP	0.4565	54.06065	1.59889	2.1314

Totals : 2536.35678 208.21805

==

*** End of Report ***
