# Supplementary material for: Performance of Olive-Pomace Oils in Discontinuous and Continuous Frying. Comparative Behavior with Sunflower Oils and High-Oleic Sunflower Oils
Source: Foods. 2021 Dec 11;10(12):3081. doi: 10.3390/foods10123081 (PMC8701874; doi:10.3390/foods10123081)
Supplement: Supplementary file 1 [file foods-10-03081-s001.zip › foods-1481554-Supplementary Material/Supplementary Figure S2. OPO2 Phenolic compounds 335 nm.pdf]

Figure S2. Analysis of phenolic compounds (335 nm) in olive-pomace oil (OPO2)

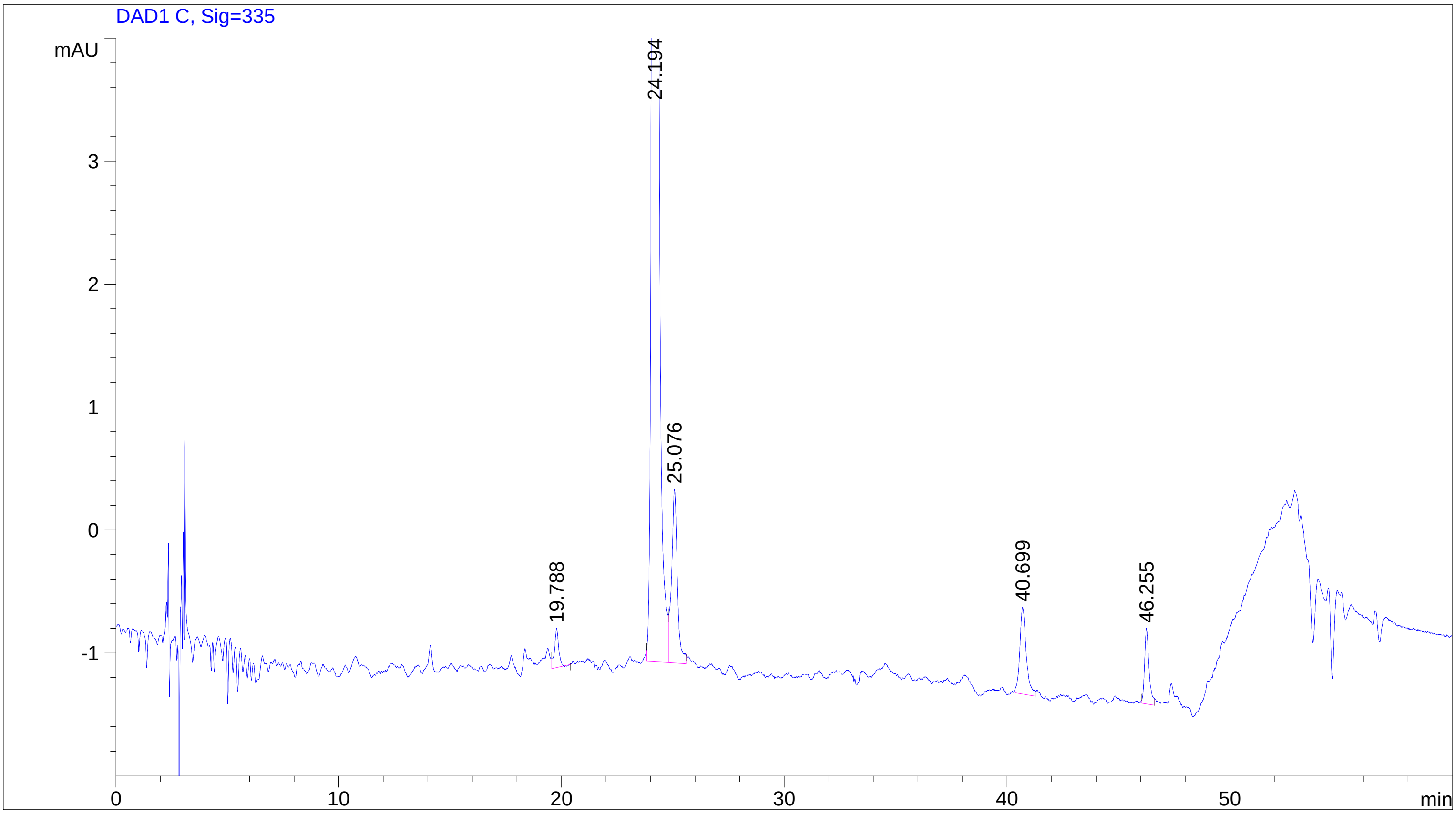

==

Area Percent Report

==

Sorted By : Signal
Multiplier : 1.0000
Dilution : 1.0000
Use Multiplier & Dilution Factor with ISTDs

Signal 1: DAD1 C, Sig=335,16 Ref=550,80, TT

Peak #	RetTime [min]	Type	Width [min]	Area [mAU*s]	Height [mAU]	Area %
1	19.788	VB	0.1914	4.13978	3.18970e-1	0.8213
2	24.194	BV	0.2093	450.87811	32.61849	89.4474
3	25.076	VB	0.2725	26.21488	1.41256	5.2006
4	40.699	BB	0.3018	14.12973	7.05096e-1	2.8031
5	46.255	BB	0.2130	8.70830	6.15853e-1	1.7276
Totals :				504.07079	35.67097	

==

*** End of Report ***
