# Supplementary material for: Performance of Olive-Pomace Oils in Discontinuous and Continuous Frying. Comparative Behavior with Sunflower Oils and High-Oleic Sunflower Oils
Source: Foods. 2021 Dec 11;10(12):3081. doi: 10.3390/foods10123081 (PMC8701874; doi:10.3390/foods10123081)
Supplement: Supplementary file 1 [file foods-10-03081-s001.zip › foods-1481554-Supplementary Material/Supplementary Figure S3. Acceptability test evaluation sheet .pdf]

ACCEPTABILITY TEST. Fried potatoes

1. Texture

Not crispy								Very crispy
1	2	3	4	5	6	7	8	9
☐	☐	☐	☐	☐	☐	☐	☐	☐

2. Oiliness

Not oily								Very oily
1	2	3	4	5	6	7	8	9
☐	☐	☐	☐	☐	☐	☐	☐	☐

3. Taste

Dislike extremely								Like extremely
1	2	3	4	5	6	7	8	9
☐	☐	☐	☐	☐	☐	☐	☐	☐

4. Color

Very light								Very dark
1	2	3	4	5	6	7	8	9
☐	☐	☐	☐	☐	☐	☐	☐	☐

5. Global appreciation

Dislike extremely								Like extremely
1	2	3	4	5	6	7	8	9
☐	☐	☐	☐	☐	☐	☐	☐	☐

Definition of attributes

- Texture is referred specifically to crispiness perception of the outer surface from the first bite and during mastication.
- Oiliness is referred to the gustatory perception of oil quantity on the surface and in the core.
- Taste is referred to the gustatory sensation in a hedonic scale expressing degree of dislike/ like.
- Color is referred to description in terms of intensity.
- Global appreciation is the overall acceptability according to the attributes evaluated and any other, in a hedonic scale expressing degree of dislike/like.
